# Intertrochanteric Fracture of the Ankylosed Hip Joint Treated by a Gamma Nail: A Case Report

**DOI:** 10.1155/2012/278156

**Published:** 2012-08-01

**Authors:** Daichi Ishimaru, Satoshi Nozawa, Masato Maeda, Katsuji Shimizu

**Affiliations:** ^1^Department of Orthopaedic Surgery, Takayama Red Cross Hospital, 3-11 Tenman, Takayama, Gifu 506-8550, Japan; ^2^Department of Orthopaedic Surgery, Graduate School of Medicine, Gifu University, 1-1 Yanagido, Gifu 501-1194, Japan

## Abstract

We herein report a rare case of an intertrochanteric fracture complicated with an ankylosed hip joint in a 76-year-old man. Generally, operative treatment is performed for elderly people with intertrochanteric fractures to prevent general complications, maintain mobility, and release pain. However, intertrochanteric fractures of ankylosed hip joints are rare, and the optimal surgery for this condition is unknown. In addition, surgical fracture repair is challenging because unusual instability of the fracture site is suspected owing to the long lever arm of the lower extremity. Nevertheless, we successfully treated this rare fracture using a gamma nail, which may be a useful implant with which to treat this type of fracture if the status of the arthrodesed hip joint allows.

## 1. Introduction

In recent years, the incidence of hip fractures has been increasing with the aging of populations in industrialized countries, and approximately 50% of hip fractures are intertrochanteric fractures [1]. In general, operative treatment is performed for patients with intertrochanteric fractures; however, there is little available information regarding intertrochanteric fractures of arthrodesed or ankylosed hip joints. To the best of our knowledge, only a few cases of proximal femoral fractures in ankylosed hip joints have been reported; these cases were treated surgically using either a plate or a retrograde intramedullary nail [2, 3]. For orthopaedic surgeons, selection of the most appropriate surgical procedure for intertrochanteric fractures of ankylosed hip joints is challenging because the muscles around the hip joint are generally severely weakened and the hip joint is mostly immobile. We herein present a rare case of an elderly patient with an intertrochanteric fracture of an ankylosed hip after failed arthrodesis. This case was successfully treated with surgery using a gamma nail despite the fact that the patient had severe hip muscle atrophy and immobility of the hip joint owing to the ankylosed hip. Gamma nails may be useful implants to obtain rigid fixation for intertrochanteric fractures, even in immobilized hip joint, as long as the herein described critical points are kept in mind. 

## 2. Case Presentation

**Figure 1:**
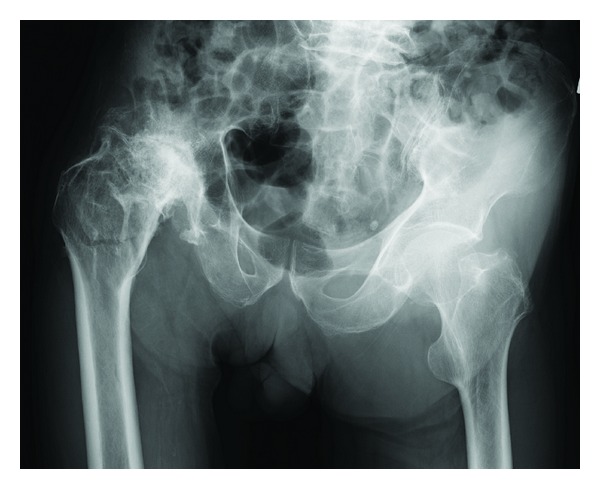
Anteroposterior radiograph showing an intertrochanteric fracture of the right hip joint that was ankylosed because of arthrodesis performed 56 years earlier.

**Figure 2:**
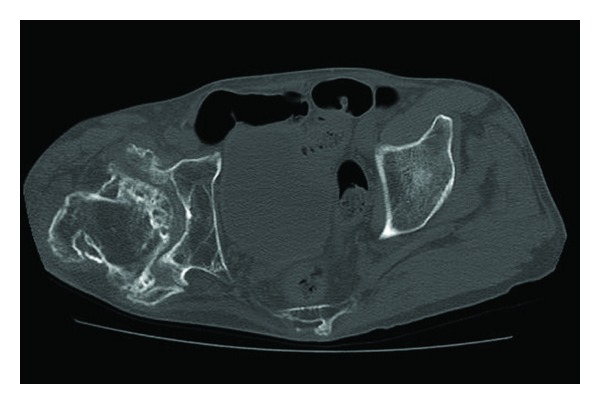
CT imaging around the hip showing marked gluteus muscle atrophy.

**Figure 3:**
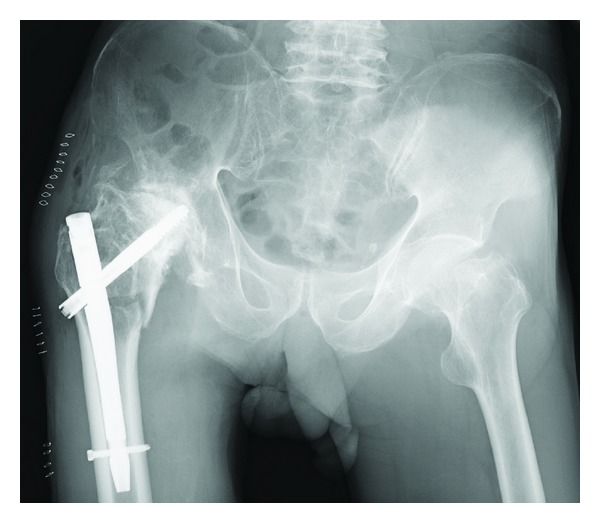
Anteroposterior radiograph captured after the operation showing the intertrochanteric fracture stabilized with a gamma nail.

**Figure 4:**
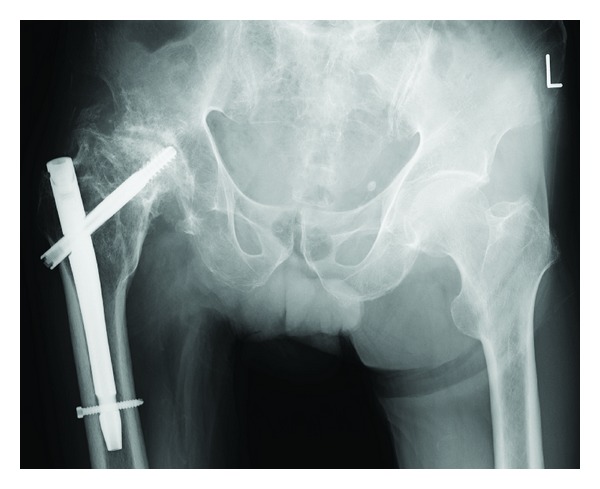
Anteroposterior radiograph captured 8 months after the operation showing strong bone union.

A 76-year-old man fell down and was transferred to our hospital. He complained of right hip pain and inability to walk. The right hip joint exhibited contracture at 20° of adduction and 10° of flexion. A plain radiograph revealed an intertrochanteric fracture and severe deformity of the right hip joint (Figure 1). Indeed, the patient had undergone hip arthrodesis surgery 56 years previously for a right femoral neck fracture, which had necessitated bone transplantation and the use of a cast for 6 months. This treatment resulted in an ankylosed rather than arthrodesed hip joint. Computed tomography (CT) imaging of the right hip joint revealed a displaced intertrochanteric fracture under the ankylosed hip joint and marked atrophy of the gluteus muscles (Figure 2). Significant ectopic ossification and osteophyte development around the hip joint was suggested to be the cause of the limitation in the range of hip joint motion at all angles. Based on these findings and the radiographic images, he was diagnosed not with a simple intertrochanteric fracture, but an intertrochanteric fracture under an ankylosed hip joint. We carefully discussed the appropriate operative procedure for this condition in detail because there are few previous cases similar to this one. Gamma nail fixation was performed 8 days after the fracture. Preoperative fluoroscopic images with the patient under spinal anesthesia showed that the right hip joint was still totally immobile and fixed in adduction at 20° and flexion at 10°. The instrument used was a gamma nail (Stryker Howmedica) (Figure 3). For 4 weeks after the operation, the right hip joint was set in a cast extending from the trunk to the right femur because there could be considerable stress at the fracture site. Partial weight bearing was allowed 2 weeks after surgery, and full weight bearing with the use of double crutches was allowed 4 weeks after surgery. One year after the operation, bone union was completely achieved, and the patient was satisfied with his ability to walk with double crutches (Figure 4).

## 3. Discussion

In this paper, we presented a case of an intertrochanteric fracture, severe gluteal muscle atrophy, and an ankylosed hip joint after failed arthrodesis. This type of fracture is relatively rare. In this case, the fracture was unstable because the hip joint proximal to the fracture site was fixed fibrously and a long distal lever arm of the lower extremity was present. Furthermore, the muscle condition around the fracture site was poor because of the long-term hip ankylosis. This case was considered to be challenging in terms of surgical treatment. Although there were many surgical options, we successfully treated this rare fracture using a gamma nail.

In industrialized countries, the number of hip fractures has been increasing with the aging of populations [1], and generally, surgical treatment is strongly recommended as soon as possible. However, debate over the optimal treatment for proximal femoral fractures around arthrodesed or ankylosed hip joints remain, and only two cases have been reported to date [2, 3]. We considered several surgical procedures in the present case, including the use of plate fixation [2], a compression hip screw [4, 5], an intramedullary nail along with a gamma nail [4, 6], or total hip replacement (THR) for the arthrodesed hip [7–9]. Plate fixation is considered unsuitable for patients with severe gluteal muscle atrophy because this procedure often requires widespread muscle detachment. Moreover, a compression hip screw would have failed to sufficiently grasp the proximal fragment and is unsuitable for early weight bearing, which were important considerations for the unstable fracture in the present case. Currently, THR for arthrodesed or ankylosed hip joints is frequently performed. However, it is technically demanding because of the lack of surgical landmarks secondary to the ankylosis, and failure rates range from 14% to 29% [8, 9]. In our case, THR was considered to be contraindicated because of the patient's age and hip joint condition, including gluteal muscle atrophy. Finally, the gamma nail was determined to be theoretically advantageous over the intramedullary nail with minimal invasiveness to muscles around the hip joint and greater mechanical resistance at the fracture site, which would allow early weight bearing. Moreover, gamma nails yield better functional results for unstable trochanteric fractures than do compression hip screws [10].

**Figure 5:**
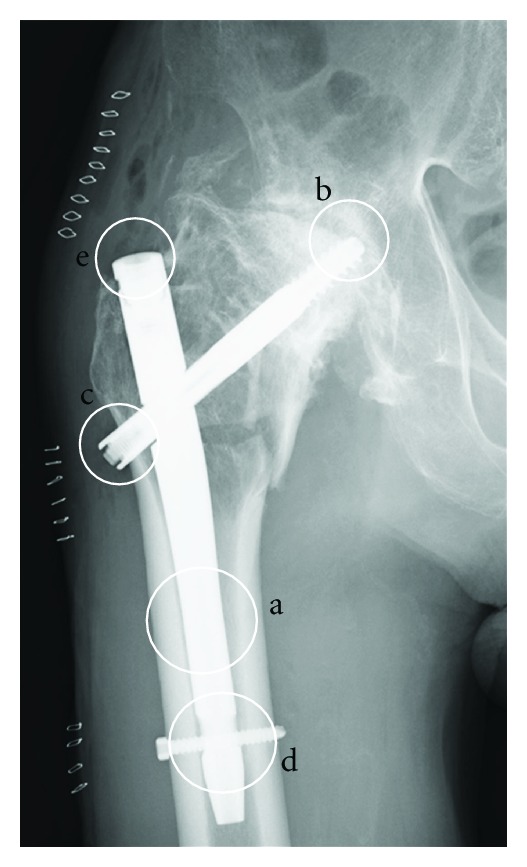
Anteroposterior radiograph after the operation showing the important points of operation for an intertrochanteric fracture of an ankylosed hip, represented with five circles (a–e).

The following points were considered for the gamma nail placement to obtain excellent fixation. First, we selected a thick nail of 12-mm diameter to fit the medullary cavity (Figure 5(a)). After investigating the original neck position of the femur from the preoperative CT scan, we precisely inserted the lag screw into the cranial side of the calcar of the femoral neck; the screw extended to the end of the femoral head whereby a tight grip was ensured (Figure 5(b)). To prevent nail rotation, the lateral edge of the lag screw was located outside the femur (Figure 5(c)), and a distal screw was inserted (Figure 5(d)). An end cap of 5 mm extension was chosen to ensure sufficient contact of the nail with the cortical bone and thereby avoid instability toward both adduction and abduction (nail, Figure 5(e)). Postoperative CT scans proved that the above procedure was successful. Using the gamma nail, we adequately fixed the intertrochanteric fracture of the ankylosed hip to allow early weight bearing, enabling the patient to recover and walk with double crutches. We did provide a cast after the operation because we were concerned that the gamma nail would break and another fracture would occur around the implant secondary to unusual stress. In this surgical treatment, there is a limitation about selecting the size of gamma nail. Concerning the thickness of the gamma nail, in Europe, diameter 12 mm of the gamma nail we selected does not exist, although a thicker nail may be useful in order to fit the medullary cavity of femur.

 In summary, we herein described a rare case of an intertrochanteric fracture with an ankylosed hip joint. Although the muscle condition around the hip joint, including the gluteal muscle, was so poor, the gamma nail was a useful implant with which to treat this type of fracture with minimal invasiveness to the atrophic gluteal muscle.
